# ‘I was just doing what a normal gay man would do, right?’: The biopolitics of substance use and the mental health of sexual minority men

**DOI:** 10.1177/1363459321996753

**Published:** 2021-02-25

**Authors:** Mark Gaspar, Zack Marshall, Barry D. Adam, David J. Brennan, Joseph Cox, Nathan Lachowsky, Gilles Lambert, David Moore, Trevor A. Hart, Daniel Grace

**Affiliations:** University of Toronto, Canada; McGill University, Canada; University of Windsor, Canada; University of Toronto, Canada; McGill University, Canada; University of Victoria, Canada; CIUSSS du Centre-Sud-de-l’Île-de-Montréal, Canada; University of British Columbia, Canada; Ryerson University, Canada; University of Toronto, Canada

**Keywords:** biopolitics, technologies of the self, ambivalence, mental health, substance use, gay, bisexual, queer and other men who have sex with men

## Abstract

Drawing on 24 interviews conducted with gay, bisexual, queer and other men who have sex with men (GBM) living in Toronto, Canada, we examined how they are making sense of the relationship between their mental health and substance use. We draw from the literature on the biopolitics of substance use to document how GBM self-regulate and use alcohol and other drugs (AODC) as *technologies of the self*. Despite cultural understandings of substance use as integral to GBM communities and subjectivity, GBM can be ambivalent about their AODC. Participants discussed taking substances positively as a therapeutic mental health aid and negatively as being corrosive to their mental wellbeing. A fine line was communicated between substance use being self-productive or self-destructive. Some discussed having made ‘problematic’ or ‘unhealthy’ drug-taking decisions, while others presented themselves as self-controlled, responsible neoliberal actors doing ‘what a normal gay man would do’. This ambivalence is related to the polarizing binary community and scientific discourses on substances (i.e. addiction/healthy use, irrational/rational, uncontrolled/controlled). Our findings add to the critical drug literature by demonstrating how reifying and/or dismantling the coherency of such substance use binaries can serve as a biopolitical site for some GBM to construct their identities and demonstrate healthy, ‘responsible’ subjectivity.

## Introduction

Determining what constitutes ‘problematic’ alcohol and other drug consumption (AODC) can be a contentious affair. For example, in Toronto, Canada, HIV activists recently engaged in heated debates on crystal methamphetamine use among gay, bisexual, queer and other men who have sex with men (GBM). Some argued that the uptake of crystal methamphetamine is dangerous and requires a firm prevention approach, while others averred that healthier patterns of methamphetamine use are possible and that the denial of this reality perpetuates stigma which ultimately harms GBM (Valelly et al., 2019). While the topic of crystal methamphetamine has elicited heightened emotional responses globally (Bryant et al., 2018), the polemical nature of these debates is indicative of a greater problematic within the GBM health sector: how to effectively address some of the noted health concerns related to AODC while avoiding a reification of stigmatizing belief systems. Critically exploring this tension, particularly from the point of view of GBM managing mental distress in their everyday lives, is the aim of the following analysis.

In the public health and biomedical literatures, AODC is often considered to be a factor exacerbating health disparities among GBM, and notably correlated to worse mental health. This scholarship posits that GBM report higher levels of AODC, substance use disorders and worse mental health outcomes (i.e. anxiety, depression, suicidality) than their heterosexual counterparts (Brennan et al., 2010; Kelly et al., 2015; Lachowsky et al., 2017). Within this literature, experiencing minority stress – distress caused by discrimination and hardship related to one’s status in an oppressed group – is thought to encourage some GBM to consume alcohol and drugs in ways that aggravate mental health problems (Feinstein and Newcomb, 2016), and discrimination, victimisation and social isolation are associated with the concurrence of substance use and psychological distress (Bränström and Pachankis, 2018). HIV stigma is also thought to increase substance taking, leading to poorer HIV treatment adherence and worse mental health outcomes among GBM living with HIV (Edelman et al., 2016).

Another approach to the study of GBM and substance use derives from the critical social sciences. Though recognising the potential links between trauma, structural violence and mental health on some patterns of AODC, these scholars have been sceptical of dominant biomedical causal narratives. They argue that focusing on AODC as ‘pain amelioration’ from trauma tends to collapse a variety of AODC practices, focuses on individual-level pathology, minimizes the function of culture, occludes the possibility for agency and rationality within drug decision-making and denies the co-occurrence of pleasure with ‘problematic use’ (Bryant et al., 2018; Pienaar et al., 2020; Valentine and Fraser, 2008). Pienaar et al. (2017) explain that as ‘a co-constituted phenomenon, addiction is made in its encounters with social isolation, marginalisation, homelessness and institutional neglect but also in the pleasure of partying, socializing, responsible work and a full life’ (p. 532).

Critical social scientists have documented the beneficial dimensions of substance use for some GBM (Pienaar et al., 2020; Race et al., 2017), demonstrating how substances can facilitate processes of self-realisation, pleasure, disinhibition, community connection and sexual desire (Hawkins et al., 2019; Souleymanov et al., 2019). They can help GBM achieve the sex they want by reducing anxieties related to homophobia, HIV or body image (Race et al., 2017). Venues where substance use is common have been important sites for social bonding, political organising and for sharing harm reduction messages (Race et al., 2017). Despite this, GBM can also stigmatize drug-taking practices (Race et al., 2017).

This stigma is partially a result of the binary logic (i.e. addiction/healthy use, problematic/non-problematic use, chaos/order, compulsion/volition, irrationality/rationality) that saturates discourse on substance use and that tends to emphasize the negative dimensions of drugs (Pienaar et al., 2017). However, ‘these binaries struggle to attend to the rich and varied health perspectives and experiences of those who self-identify as living with an addiction, dependence or habit’ (Moore et al., 2017: 156). The tendency toward dichotomous thinking on substance use may account for the polarizing community debates about GBM and Party n’ Play (PnP)/chemsex (i.e. sex with polydrug use, typically involving crystal methamphetamine). Given the impetus to demarcate AODC as either healthy or unhealthy, non-problematic or problematic, there is less discursive space to account for the complexities of AODC among GBM.

Our analysis takes up this complexity. Rather than reinscribing a biomedical narrative articulating the causal pathways to ‘problematic use’, in this paper we draw from the literature on the biopolitics of substance use to document the multifarious and contingent means by which GBM self-regulate and draw on AODC as *technologies of the self*. While the critical social scientific literature on GBM has tended to focus on the interrelationship between AODC, sexuality and sexual pleasure (Pienaar et al., 2020), less attention has gone into examining the variable meanings GBM draw on to make sense of the interplay between their mental health, emotional wellbeing and their AODC.

### The biopolitics of substance use

Pienaar et al. (2020) draw on Foucault’s concept of *technologies of the self* to explain how sexual and gender minorities have used drugs to enhance their experiences of sex, sexuality, gender and pleasure. With *technologies of the self*, Pienaar et al. focus on how drugs allow individuals to enact ‘new subjectivities, particularly ones that depart from intelligible, disciplinary forms’ (p. 2). This analytic approach grants sexual and gender minorities agency in their drug-taking, rather than *a priori* pathologizing their use. Pienaar et al. (2017) have argued that a reliance on the biomedical disease model to describe AODC ‘often constitutes those considered to be experiencing addiction as lacking free will and agency – both key attributes of the “proper” neoliberal subject. This enactment renders them flawed citizens by virtue of their presumed inability to exercise autonomy and self-control’ (p. 532). Fraser et al. (2017) have similarly argued that substance use stigma operates as a ‘performative biopolitical technology of power, one that constitutes the very conditions under which legitimate subjects emerge’ (p. 194). They clarify that ‘the outside of legitimacy comprises all those whose relationship to drugs does not align with normative understanding of autonomy, sobriety, freedom and rationality’ (p. 195). The management of AODC does not merely determine who is a ‘failed’ neoliberal subject, but also creates the conditions for the ‘healthy’ neoliberal subject to materialize. Thus, in addition to drawing on drugs as technologies to enhance pleasure, people can also draw on dominant biomedical discourses about drugs and ‘problematic use’ as *technologies of the self* to establish or ‘perform’ healthy subjectivity.

These observations align with Foucauldian biopolitics. Foucault (1978/1990) presents biopolitics as a form of power invested in fostering life or disallowing it (to the point of death). Investment in the optimisation of life through discourses of ‘healthy’ or ‘responsible’ AODC has as its counterpart the generation of the stigmatized, unhealthy other. Societal attention to encouraging ‘healthy’ substance use practices can be envisioned as ways in which subjectivity is produced and bodies that consume alcohol and illicit drugs are governed (Fraser, 2004). Governmentality scholars have well established how under neoliberalism, disciplinary power has led to the rise of ‘health conscious citizens’ who are ‘autonomous individuals wilfully regulating themselves’ (Ayo, 2012: 100). Articulating oneself as living healthily is never value-neutral, but is a moral project whereby socially desired ‘values such as prudence, hard work, responsibility and asceticism are expressed’ (Ayo, 2012: 101; Pienaar et al., 2017). GBM have long self-regulated their sexual behaviours to demonstrate neoliberal principles of responsibility, autonomy, self-control and rationality in the face of HIV risk and stigma (Adam, 2016).

Hence, a biopolitical analysis of substance use attends to how individuals generate subjectivity in and through health discourses in ways that have both productive as well as repressive/disciplinary effects. In the analysis below, we focus on how GBM draw on complex discourses of mental health and emotional wellbeing to make sense of their AODC, to self-regulate, and establish identity.

## Methods

We conducted 24 in-depth interviews about mental health with GBM living in Toronto. These interviewees were recruited from a list of those who had completed a quantitative survey and biomedical screening from the Engage study. Engage is a respondent driven-sampling study taking place in Montréal, Toronto and Vancouver, which focuses on the health of sexual minority men, including their sexual health, mental health and substance use. Recruiting from Engage allowed us to consult with the Community Engagement Committee (CEC) in Toronto. The CEC was made up of GBM and GBM healthcare providers who contributed feedback on the study design, interview guide, analysis plans and community knowledge dissemination. With the CEC, the key objectives of this qualitative mental health study were finalized. These objectives included: HIV biomedical advances and mental health (Gaspar et al., 2019); mental healthcare access experiences (Gaspar et al., 2021); substance use and mental health (present analysis) and sexual coercion and mental health (analysis forthcoming).

Prospective Engage participants who had previously provided consent to be contacted for additional studies were emailed and asked if they would be interested in contributing to a qualitative study about mental health. We interviewed men with diverse mental health experiences including those dealing with milder degrees of distress, and those with more severe clinical diagnoses. The study documented how many GBM wanted access to mental health supports, but faced significant challenges receiving affordable, culturally competent mental healthcare, which provoked further distress among a group already dealing with significant social and economic precarity (see Gaspar et al., 2021). Understanding substance use practices and experiences with health services for AODC were underscored as part of this study’s objectives. We did not recruit for specific AODC patterns or only for people who self-identified as having an addiction, dependence or habit. Though a potential limitation, this also allowed for a diversity of AODC experiences within our sample. Ethics approval was granted by the University of Toronto, Ryerson University and the University of Windsor.

The one-on-one interviews took place in-person at the University of Toronto and were conducted by the first author. Participants provided written informed consent before the interview. An interview guide was used. After introductions and socio-demographic information, participants were asked about their mental health history and experiences with mental healthcare. Participants were then asked to describe their AODC and the impact – positive and/or negative – of substances on their mental health. We also asked if they have ever spoken to a healthcare provider about their AODC. Additionally, interviewees were questioned about their experiences managing their sexual health, HIV, other sexually transmitted infections and sexual coercion.

Instead of relying exclusively on the interview guide, an active approach to interviewing was used. This open-ended method gathers data by following a participant’s conversation carefully to acquire information in an organic fashion (Holstein and Gubrium, 1995). The interviews ranged from 40 to 120 minutes. They were audio recorded and transcribed verbatim. Participants were given $30 CAD and information about available mental health services. We identify the participants below using pseudonyms.

The transcripts were uploaded into NVivo 11 software and analysed using Charmaz’s (2014) articulation of grounded theory, which prioritises an inductive analysis of collected evidence first, before consulting extant theories. As Shuster (2016) articulates, this modified grounded theory then comprises ‘a recursive process of moving back and forth between the raw data, coding schema, and literature with the goal of further refining codes into subcategories and looking for patterns in the data’ (p. 323). In practical terms, first the interview transcripts were coded line-by-line for all pertinent themes. This built familiarity with the complete dataset and helped to build an understanding of the particularities of each participant’s account. Second, our analysis focused on sequentially examining the substantial topical areas of the dataset (i.e. which reflect the four main study objectives outlined above) to note major patterns. During this stage, we observed that stigma and ambivalence were reoccurring themes in the raw data on substance use. The significance and generalizability of these themes to interpret the dataset were then examined by closely reading each participant’s reflections on their substance use. In the final ‘theoretical coding’ stage of analysis, we iteratively engaged with the outcomes of stage two and the relevant literature and theory discussed above to finalize the results.

## Findings

Of the 24 participants, 22 (92%) identified as gay and/or queer and 2 (8%) as bisexual. One (4%) identified as gender non-binary and 1 (4%) as a trans man. The average age was 37 years old (range: 22–59). Fourteen (58%) identified as White, 2 (8%) as Latino, 3 (13%) as African, Black or Caribbean, 3 (13%) as East or South Asian and 2 (8%) as Middle Eastern. Half of the sample (50%) reported annual incomes of $40,000 CAD or lower. Fifteen (63%) participants identified as being HIV-negative and 9 (38%) as people living with HIV.

Three (13%) participants described having used only alcohol and cannabis. The majority (87%) detailed having used other drugs. Seven participants (29%) described frequently using party drugs (e.g. MDMA, cocaine, GHB) and participation in the PnP/chemsex scene. Two participants (8%) identified as not currently drinking or using drugs. Seven participants (29%) discussed having previously used crystal methamphetamine, with two men (8%) having taken it intravenously. One participant (4%) reported crack use. No participant described taking heroin or issues with opioids. Five men (21%) described more significant health challenges linked to AODC with three (13%) of these men terming their experiences as ‘addiction’ or ‘substance abuse’.

### Mental wellbeing and substances as complex technologies of the self

When participants were asked to describe the relationship between their substance use and their mental health, many explained how taking drugs had positive effects on their mental wellbeing and could be used as *technologies of self-care*. For example, some described using cannabis for their anxiety or post-traumatic stress disorder. Claudio (20s) explained how he was ‘micro-dosing’ LSD for his attention deficit disorder. Several detailed combining prescription drugs with other drugs to manage their mental health, including Ang (30s) who mixed cannabis with antidepressants. Ang also discussed how he used MDMA, mushrooms, and LSD for his mental health: ‘It helps me get like an altered perspective on my own mental state and my emotional state and just to check in with myself and work through some of the feelings that I have’.

Bradley (30s) detailed how he ‘had a blast’ taking drugs like MDMA at Gay Pride. However, with his chronic depression and anxiety in mind, he noted how with drugs ‘the cycle of getting high and coming down is not one of stability’. As a transman who recently underwent gender confirming surgery, he stated that: ‘I just needed to be in the best health ever in order to like, heal well from that. In terms of MDMA, you do enough of that stuff, it’s really going to deplete your serotonin levels’. Mitch (50s) likewise discussed how he used to love going to gay circuit parties but stopped taking drugs because it ‘made me crazy’ and the comedown from ecstasy, which he labelled as ‘suicide Tuesdays’, was ‘just terrible. I’m like in tears and it’s horrible’. While John (30s) expressed similar concerns: ‘it’s something that kind of scares me now ‘cause I don’t know if I feel like, I’m not mentally strong enough to endure like, the comedown off some of these drugs’. Bill (20s) explained that he was curious to try LSD and shrooms, but worried about his mental health since alcoholism ran in his family and his father died from suicide.

Participants thus positioned drugs as complex *technologies of the self*, as tools aiding self-realisation, emotional self-monitoring and self-care, as well as tools to establish gay identity, seek pleasure and social connection. They also drew on discourses of mental health and emotional wellbeing as *technologies of the self* – for example, hormones, the genetic basis of addiction, antidepressant technology, clinical diagnoses – to assert agency and a sense of rationality over their AODC.

### Ambivalence and self-regulating use

Many participants discussed closely monitoring their AODC and reducing or planning to reduce their intake to instigate more ‘ideal’ or ‘healthier’ use – the hallmark of biopolitical self-regulation (Ayo, 2012; Pienaar et al., 2017). These participants were often highly critical and ambivalent about their use. For example, Kyle (30s) judged his drinking patterns as such: ‘I do drink too much. That’s a self-assessment which I’m working with and [drinking] helps if I’m having a bad day and if I can’t reach out to a friend. And if I do [drink] sometimes, that’s an interim solution which I recognize is not ideal’. Riaan (30s) also paid active attention to his drinking. He stated how alcohol was an important part of his social life: ‘I don’t want to be the person who gives up on alcohol because I still like its social lubrication. It’s part of my culture here’. However, he also discussed how ‘after a night of drinking, I will feel what depression feels like’. Riaan described relying on alcohol during his medical residency: ‘Was it the healthiest? No. Like you know, it definitely had its side effects in terms of things like the hangover and then not really dealing with the emotions you’re feeling and just trying to dull them with alcohol’. Riaan discussed struggling to come to terms with his sexuality during this period. Collectively, this was a difficult moment where alcohol was used as an ambivalent therapeutic aid: ‘There was so much fear and anxiety and stress that I would use alcohol to get it out. So, there was a therapeutic aspect, but also a very kind of destructive aspect to it as well’. Both Kyle and Riaan invoked the dichotomy of healthy/unhealthy drinking. However, by describing the practical benefits of drinking for their mental health as an ‘interim solution’, they also challenged this binary’s capacity to adequately explain the complex role of alcohol in their lives.

Luis (40s) similarly talked about formerly addressing his depression with alcohol as a way ‘to destruct myself’, which lead him to stop drinking. He described how his ex-husband used to stigmatize his use by making him feel ‘guilt and shame’ for ‘being a pothead’ even though Luis was using cannabis for his anxiety and post-traumatic stress disorder. Luis then recounted a period where he was interested in PnP as a form of queer self-discovery after his divorce and being the ‘perfect husband’. However, he explained how slamming (i.e. injecting) methamphetamine felt challenging to his sense of identity: ‘I think I went too far. I don’t see myself like injecting something and I mean, that’s not me’. Drawing on the trope that certain patterns of drug use represent an inauthentic version of one’s character (Moore et al., 2017), Luis’ simultaneously presented methamphetamine as a *technology of self* to seek his identity and as a potential threat to his sense of self.

Throughout his interview, Ali (30s) discussed struggling with anxiety and panic disorder. He then described his crystal methamphetamine use as such: ‘I have noticed with this whole [recent] job loss thing that my emotions are not in my control, sometimes to escape I will use more hardcore substances than easier substances. I have dabbled in meth’. Ali clarified that even when taking methamphetamine ‘I like to be in control’ and that he ‘could not physically lose myself in it’. He was critical of men using crystal methamphetamine who acted out of control: ‘I just think it’s a crock of shit when people have symptoms, it’s embarrassing and then I throw them out’.

Ali’s comments resonate with the critical drug literature by demonstrating how the concept of *self-control* may be used to communicate a healthier (i.e. non-problematic) attachment to drugs in order to distinguish one’s use as more ideal than others (Fraser et al., 2017; Pienaar et al., 2017). However, Ali’s reflections that he was taking more ‘hardcore substances’ during a period of heightened emotional duress indicates an implicit recognition that his drug-taking may be more compulsive than that which is aligned with his identity as a controlled person. Ali simultaneously framed his methamphetamine consumption as a form of escapism from uncontrollable feelings caused by unemployment, and communicated how much self-control he had over his drug experiences (i.e. to the point where he could not escape and enjoy the experience). Ali’s ambivalence with crystal methamphetamine is indicative of how binary discursive frames can be insufficient for describing the motivations for taking substances, where ostensibly opposed values, such as control and uncontrollability, actually operate co-constitutively. This dynamic was also observable when he discussed how heightened negative attitudes toward crystal methamphetamine often manifested through an immediate pathologization of occasional use:

Once in a while [I do meth]. I’m not dependent on [it]. I notice that this country is the only one where substance use becomes like, ‘Oh my god, it’s an addiction!’ [. . .] Oh he took it once! That’s it! He’s addicted to meth—I’ll put him in a treatment centre!

According to Ali, the polarizing binary discourse on crystal methamphetamine creates a context where there can be no pattern of use that is not considered inherently problematic. Hence, if someone like Ali does not subjectively envision himself as a ‘meth user’ (he ‘dabbles’) or an ‘addict’ (‘I’m not dependent’), there is limited discursive space for him to ask questions regarding the interplay between his crystal methamphetamine use and emotional wellbeing, without automatically pathologizing his behaviour and/or realigning his sense of identity.

Several participants mentioned that there were certain drugs – in particular, crystal methamphetamine, crack, heroin and taking drugs intravenously – that they would never do. Such distinctions reified the division between ‘ideal/non-ideal’ forms of AODC. John (30s), for instance, vocalised discomfort with methamphetamine use: ‘I see people like, in the [GBM] community doing meth and things like that, and it scares me . . .. It just makes me uncomfortable’. While Bill (20s) stated that he was surprised by the number of GBM ‘who seem to have a level head’ that he knew who were using cocaine: ‘it’s just baffling [. . .] the amount of people that I know who do coke who aren’t what you would imagine being cokeheads’. Such examples of stereotyping over the amount and/or type of substances consumed were common across the sample, though more often implicit than explicit. For instance, John considered drugs a thing of the past and something that one grows out of: ‘I’m not that interested in [drugs] I guess, I don’t know. I just, it’s not something that really interests me. To do drugs now, like, I still have friends who are in their mid-30s who are still doing party drugs’.

Meanwhile, Jose (30s) recounted how he started using more drugs in the context of a relationship. He described how this former partner ‘gave me so much anxiety, cause he was so irresponsible. And things were wild, like, I was never exposed to drugs besides marijuana and like, he did it all’. Jose framed his drug experiences as experimental:

I tried it all, didn’t touch meth or crack. And it was just experimental, like, I never like, felt like oh, I need it. I was just experimenting. I was doing things more recklessly and . . . and I kind of felt like I had to [use drugs], like, ‘cause I had so much anxiety about not being social enough. I was just doing what a normal gay man would do, right?’

Jose thus simultaneously held his former ‘irresponsible’ partner accountable for ‘exposing’ him to drugs, while also demonstrating a heightened sense of agency over his AODC. He clarified that he did not use more maligned drugs like methamphetamine or crack cocaine, thus distinguishing his substance use history as more ideal. He described his former AODC as a tool that helped him overcome social anxiety as a newly out gay man. By clarifying that he was in control of his drug use and never dependent (‘I never like, felt like oh, I need it’; ‘I was never like that’), and that it was temporary (‘just experimenting’), he framed his drug-taking as more socially acceptable. Moreover, by stating that this is just what ‘a normal gay man would do’, Jose drew on cultural perceptions of GBM’s AODC to recast any risks as normative (Race et al., 2017). Nonetheless, Jose also described his behaviours during this period as ‘reckless’ indicating some self-judgement.

When discussing his MDMA use, Don mentioned that ‘I used to have a lot of anxiety about those, and actually, marijuana too. I grew up in the States during the War on Drugs, so I was scared to death of anything like that’. However, he described that while he had previously been ‘turned off by my own nervousness’ he now approached drugs with a ‘very sceptical curiosity’. Don’s account is illuminating for positioning his concerns with drugs and associated mental distress as a product of the illegality of substances and anti-drug discourse. Ambivalence regarding AODC’s relationship with mental health can thus arise from navigating the cognitive dissonance (i.e. ‘sceptical curiosity’) of desiring drugs within a broader social context that has historically maligned and criminalised such desire.

These participant accounts demonstrate how some GBM are highly reflexive about their substance taking, but can have ambivalent feelings regarding their AODC and its relationship to their mental health. On the one hand, AODC was positioned as fun, a way to address social anxiety, and something therapeutic. On the other hand, participants described substances in negative terms (i.e. ‘reckless’, ‘destructive’). AODC was both an important tool to forge GBM identity and new social connections, and a potential threat to one’s identity. It was both a tool to address mental distress and a potential source of mental distress. AODC was a way to escape, experiment and liberate oneself, and something that needed to be tightly controlled. This ambivalence – and with it, the impetus for self-critique, or explicit and implicit judgement and stigma toward other people’s AODC – is arguably related to the polarizing structures determining discourse on substances (Pienaar et al., 2017). In the absence of understanding the co-constitutive nature of negative and positive dimensions of use, the biopolitical drive to ‘problematize’ AODC and to construct one’s subjectivity as moving toward ‘healthier’ use by demonstrating a rational and controlled disposition to substances, can flatten out the complexity of these men’s lived experiences.

### Pathologizing accounts

While the above participants described ambivalence toward their substance taking, the following GBM more explicitly pathologized their AODC. Oliver (50s) discussed struggling with substances – ‘let’s go through the alphabet: coke, crack, crystal, pot, G, K, never used heroin or anything like that’ – and expressed shame and internalized stigma over his AODC in ways that overlapped with negative perceptions around gay sex and HIV: ‘I think starting through the drug stuff and all that when I got into it, it’s reflecting on [how] I’m a dirty person. I can’t be in a loving relationship because I’m so damaged goods, used by so many people’. He talked about not drinking or taking drugs for most of his life, because ‘I was anti all this stuff, very anti. Not judgmental, it’s just not for me because [even] at this point in time, still no matter what’s going on I’m still the top of the tier. I still take care of everybody’. He described how he started doing drugs after he was introduced to them by a friend:

An acquaintance of mine used to smoke pot ‘cause he used to like to suck me off all the time. And then he introduced me to. . . I think it was crystal or blow, I don’t know. But once when I got into crystal. . .I’m not like all these people. I’m not wiggy [i.e., uncontrolled, weird]. I’m just never like that. I was having parties all the time. Wasn’t having sex because I was on the computer trying to find more people while everybody was there having sex and using all my drugs.

Oliver also recounted dealing with mental illness (i.e. bipolar disorder, multiple personality disorder, suicidality), a history of harmful psychiatric treatment, being raped as a child and again as an adult, and struggling with internalized homophobia and ‘church-type morality’. He described using drugs as a form of ‘punishing’ himself and engaging in PnP during ‘a withdrawn state of myself, very depressed’. Oliver was diagnosed with Staphylococcus, syphilis, hepatitis C and HIV. He discussed how these diagnoses – coupled with shame related to his drug use and sexuality – led him to use more: ‘I did the most amount of drugs I’ve ever done trying to kill myself pretty much’. Oliver went to a treatment centre that he found beneficial. However, he described how the ‘aftercare was disastrous’ with people discussing their AODC being a trigger for him, and prompting him to start taking drugs again. Though Oliver was now sober, he stated that: ‘I’m disgusted by it [i.e. drugs] so bad and now I’m dealing with the guilt’.

Azim (30s) similarly described experiencing clinical depression, being physically, sexually and emotionally abused, being ostracised by his parents for being gay and dealing with socioeconomic precarity as a refugee. He discussed a period where instead of ‘trying to kind of kill myself, like, you know, like quickly, I was just going to just kind of disappear into a void. So, I started seeking out just more sort of dangerous ways of engaging sexually’. Azim participated in PnP and smoked methamphetamine in part because he had hoped to die of an overdose: ‘maybe if I just kill myself slowly, maybe that’s the way to do it. [. . .] I thought maybe I would have an overdose first’. He was then diagnosed with HIV, which he described as difficult to ‘psychologically’ accept as it was tied to this period of ‘methodical self-destruction’. Azim also discussed taking drugs to explore his sexuality: ‘I enjoyed sort of the sex or the way I felt about myself when I was doing drugs. It sort of allowed me to be more uninhibited and to feel less shame around my body’. Drugs thus operated productively as *technologies of the self*, allowing Azim to explore his sexuality and overcome shame around his body – albeit only temporarily as he also stated that he ‘want[s] to learn how to have sex sober’ and that ‘I still don’t like having sex’.

These two accounts demonstrate how GBM can draw on discourses of AODC as a route to ruin and misery (Moore et al., 2017). While not denying that Azim’s and Oliver’s sex and drug decision-making were linked to their experiences of trauma, violence and mental health, from a critical biopolitical perspective, these cases also reveal how GBM can draw on a trauma narrative as *technologies of the self* in order to explain their lived experiences and establish subjectivity. However, as Valentine and Fraser (2008) remind us: ‘Associating problematic drug use with trauma and a fractured self can easily shift to a reinscription of users as deficient; where problematic drug use represents proof of trauma and nothing else’ (p. 411). This is especially apparent in Oliver’s account, where he narrates his life story through a sense of irreparable deficit. Oliver demonstrates the mental health consequences of substance use stigma (itself intersecting with other forms of prejudice like HIV stigma) and exemplifies the prominence of neoliberal virtues of responsibility and health consciousness. He distanced himself from other people who use drugs (‘I’m not like all these people’) and clarified that though he was doing drugs in ways that he perceived as problematic, this did not prevent him from being a responsible, caring person (‘I’m still the top of the tier. I still take care of everybody’). This example highlights how dominant binary thinking on substance use (i.e. responsible/irresponsible) does not discursively allow for one to simultaneously use drugs and be considered a compassionate, trustworthy person.

Oliver and Azim did not centre a *desire for drugs* in their accounts. Azim described an ambivalent interest in smoking methamphetamine as it would often make him feel sick. Oliver discussed using drugs more to attract people: ‘I don’t have addictions issues whatsoever ‘cause I’m too structured, which I found I really don’t have addiction issues. It was just . . . I hate it [i.e. drugs] and I was just doing it to have people around me, pretty people’. This may reflect these participants’ desire to avoid identifying themselves as ‘addicts’ or they may have genuinely not found (or wanted to admit to experiencing) drug-taking as pleasurable. Regardless, these men’s drug-taking was discussed more as being related to their search for queer social connection and self-discovery, rather than the desire to be high *per se* (Green, 2003).

Analogously, Ross (20s) discussed how he started taking crystal methamphetamine when he first began hooking up with men for sex online and was discovering his identity as a gay man. He described how initially ‘I didn’t really understand what it was’ but soon it quickly ‘got heavier’. He expressed repugnance toward methamphetamine:

I’ve gotten myself away from that disgusting drug. I’ve kept myself in a positive area, I’ve surrounded myself with positive people and I’ve kept that mindset. Because you can look at life in two ways when you’re in a shitty situation: glass half full or half empty. It’s your choice. Addiction is really simply a sham and it’s not so related to mental health as. . . it’s [also] who you surround yourself with.

Ross reported concealing his crystal use from his family, a principal marker of stigma. He eventually found out that he had HIV and syphilis. Ross was initially shocked by his HIV diagnosis because ‘I knew that there was a lot of stigma. I felt like I would be made fun of in the [GBM] community for it or put down’. He described how this HIV stigma led him to use more methamphetamine and fall ill: ‘Because I was so upset about it [his HIV diagnosis], and that was probably the stupidest thing ever, cause I got even sicker as I was using it [i.e. methamphetamine]’. Ross was then evicted from a homeless shelter for consuming methamphetamine. He tried treatment, but once was locked in a waiting room for hours and faced insurmountable barriers to access:

When I did have my addiction and it was severely, severely bad, and I didn’t know what else to do, I tried to go to [a hospital] for help. They threw me out and said oh, call the detox centres. I called the detox centres: no beds available.

Ross explained that he stopped using methamphetamine after he started injecting it and ‘realized that I had hit rock bottom and if I continued, I would be dead. And I would lose everything. My money was going, my friends were leaving me. My family wouldn’t talk to me’.

Like Oliver and Azim, Ross’s account also draws on a pathologizing narrative on AODC, where social vulnerability and trauma set the stage for problematic drug use, leading to self-destructive ruin and ‘hitting rock bottom’ (Moore et al., 2017). His account differs, however, by actively centring crystal methamphetamine’s addictive traits (i.e. the desire to do drugs). Ross’ narrative simultaneously relied on the discourse of addiction as a *technology of the self* to explain his behaviours, while also dismissing the credibility of addiction, classifying it as a ‘sham’, discrediting its relationship to mental health, and clarifying that how one responds to adversity is ‘your choice’. As such, he adopted dominant neoliberal discursive framings that places responsibility on the individual for experienced adversity. Thus, while critical scholars have averred that the disease model of addiction can be stigmatizing by removing agency and pleasure from drug-taking (Valentine and Fraser, 2008), Ross’ explicit rejection of the disease model arguably also reproduces a stigmatizing narrative by predominantly emphasizing individual choice and personally accountability.

Another participant, Aaron (40s), detailed a ‘wonderful relationship’ that involved drug use and partying in the gay circuit scene. Aaron’s boyfriend was eventually deported, greatly impacting Aaron’s mental health. Aaron then discussed initially taking crystal methamphetamine to stay awake as his HIV medications made him drowsy, and then participating in PnP: ‘And then, I think drug use really crept up on me, then it was a horrible breakup and nastiness. And then I ended up using crystal meth, binging, like sort of a weekend user, every second weekend user, for about 7 years’. He stated that ‘I don’t think I was dealing with [the] emotional psychological issues at that point. I was covering them up with crystal meth use’.

Aaron described being homeless and couch surfing, and being sexual assaulted during this time. He explained his drug use as ‘shameful, it was hidden’, which caused him to cease going to his doctor: ‘I just stopped taking [HIV] medications, cause I didn’t want to go see my doctor. I didn’t want to go explain what was going on’. Aaron accessed treatment for AODC but found that it ‘was not particularly helpful. [. . .] So after that, it was still a little while of using’. For most of this period, Aaron described himself as ‘a functional meth head’ capable of maintaining a job, until he was laid off during the recession that started in 2008. He described his decision to stop using methamphetamine: ‘I was like, kind of trying to get a new job and figure what was—things really changed really rapidly. And I started taking care of myself. Stopped using, as I said earlier, started volunteering, and that was like a responsibility. I had to do things’.

Meanwhile, Ben (30s) described the interconnected but discrete nature of his AODC and his mental health (i.e. depression and anxiety):

They’re all sort of the same package. But I know, for example, if I were to stop doing drugs, I’m sure I would [still] have, you know, [mental health] problems in my life. So, I don’t think – I mean, I think it’s [i.e. cocaine and drinking] a big contributor, but I don’t think it’s. . . I can’t say it’s all substance abuse that’s causing [all my mental health issues].

Like Aaron, Ben recounted how over time his partying increased and ‘addiction just kind of creeps in’. Ben noted that ‘Sometimes it’s [i.e. cocaine use] with a sexual partner. Sometimes I’ve done it alone, I’ll admit that’. He described how ‘I’ve had to call in sick to work. Just general bad behaviour is probably the best way to put it’, and how the financial repercussions associated with his drug-taking produced ‘feelings of guilt, sadness’, which made him feel like he was ‘not going anywhere with my life’. Ben avoided group therapy sessions for his AODC: ‘I just don’t really want to go out and say to the world, okay, I have a cocaine problem, here I am’.

These last two accounts are notable for demonstrating how neoliberal notions of productivity and discipline tied to one’s capacity to work and maintain financial security can be used to problematize AODC and generate subjectivity. Ben in particular expressed shame for not managing his finances differently. In both cases, the participants connected their AODC with their mental health, but they did not completely reduce their drug-taking to their mental health. While these two participants problematized their AODC through the lens of addiction or ‘substance abuse’, unlike Oliver or Ross above, they did not disparage drug-taking *per se* or negate the social and sexual benefits they received from AODC and partying. Instead, they described complex stories filled with social and economic vulnerabilities, where drugs were used to manage relationships, stress and pleasure, but where addiction slowly ‘crept in’. This metaphor of ‘creeping in’ characterized problematic AODC as being driven by an external, almost irrepressible force, but also in a gradual, observable way, indicating that these men were making deliberate choices in response to their circumstances and desires. Thus, while both Aaron and Ben reiterated stories that demonstrated how social and economic precarity can inform mental health and AODC, they did not render themselves agentless, irrational or uncontrolled.

## Discussion

Biopolitically, the interviews revealed the co-production of different GBM subjectivities and forms of self-regulation in relation to AODC. Much like with HIV risk (Adam, 2016), these GBM communicated themselves as health-conscious neoliberal risk calculators reflexively weighing the benefits and harms of substances in their lives and regulating themselves accordingly. Some incorporated substances regularly into their everyday lives as a form of mental health management, others avoided or stopped using substances altogether or readjusted the types of substances they used to better align with their mental health needs (e.g. eliminating alcohol), and some closely monitored their use in relation to their mental wellbeing.

Some participants presented themselves as having made ‘non-ideal’ or ‘problematic’ choices related to AODC while others actively communicated their responsibility, self-control and rationality, explaining that they were just doing ‘what a normal gay man would do’. Discourses on AODC thus served as a site for the neoliberal sexual subject to demonstrate their moral capacities to responsibly enact (mental) health. Those who consumed drugs infrequently or those who had stopped taking drugs often used rhetorical tactics to distance themselves from their use, framing it as experimental, immature, in need of vigilant monitoring, or out of character behaviour. This suggests that neoliberal notions of responsible and healthy AODC can clash with socio-cultural expectations that GBM should use alcohol and illicit drugs as a way to connect (socially and sexually), forge queer identity and resist the confines of heteronormative society through subversive behaviour.

The data confirm how some GBM can use alcohol and drugs as *technologies of the self*, as tools to build subjectivity, construct relationships, to seek pleasure, have sex and to manage mental distress (Pienaar et al., 2020). Participants’ experiences with AODC were often understood in relation to GBM community and cultural events, specific GBM sexual encounters and sexualised spaces (i.e. technological sex apps, PnP), and coming out. Nonetheless, despite a broader cultural understanding of substance use as integral to GBM communities and subjectivity (Race et al., 2017), GBM can be ambivalent about their AODC. On the one hand, the participants spoke about the use of substances to manage social anxiety, to have sex confidently, and to experience social connection, self-realisation and pleasure (Hawkins et al., 2019). On the other hand, participants spoke about AODC as corrosive to their mental wellbeing and to their relationships. Some participants simultaneously described their drug use as something they actively desired, and as a response to broader social expectations and pressures among GBM to do drugs. Others concurrently discussed participating in PnP as a way to connect socially and build sexual confidence, and as an explicit avenue toward self-harm, suicide and a ‘covering up’ of mental and emotional unwellness. There was often a fine line communicated between substance use being considered self-productive or self-destructive. Many participants simultaneously reiterated the binary between problematic/healthy substance use, while also deconstructing this binary’s capacity to satisfactorily explain their specific relationship to AODC. These findings add to the critical drug literature (Moore et al., 2017; Pienaar et al., 2017), by demonstrating how reproducing and/or dismantling the coherency of AODC binaries can serve as a biopolitical site for some GBM to enact their identities.

Several participants who said they had struggled with their substance taking situated their experiences within broader stories of social and economic vulnerability, institutional violence and trauma. The belief that one’s life as a GBM is inherently worthless and inevitably prone to harm, informed how some participants discussed their motivations to use drugs with suicidal intent. Arguably, these accounts resonate with narratives found in the biomedical literature on problematic use and minority stress among GBM. Nonetheless, the participants never explicitly or exclusively attributed their drug-taking to the effects of structural violence and trauma. Instead, their accounts demonstrated how problematic AODC may operate as both a response to social and institutional harms and as way to forge social connections and enact pleasure (Moore et al., 2017; Pienaar et al., 2017). Following the critical literature, the accounts demonstrate that it is not drug-taking in and of itself that produces harms or pleasures, but an ‘assemblage’ of intersecting social, cultural, political and economic forces that enable health experience (Moore et al., 2017). Social scientists have been rightfully critical of dominant biomedical narratives on GBM drug use which pathologize drug-taking and deny pleasure and agency through trauma narratives (Bryant et al., 2018; Pienaar et al., 2020; Valentine and Fraser, 2008). However, it is also important that a focus on agency and pleasure in drug-taking does not occlude a recognition of, and interventions to then address, the social conditions and structural forces harming GBM, including among such intersecting groups like racialized refugees, men facing economic precarity, men living with HIV and men dealing with significant mental health issues.

Since biomedical discourse around GBM health has so often reproduced stigma (related to drugs, HIV and queer sex), being able to address the interaction between the benefits and harms of AODC without reifying stigmatizing understandings of drug use and denying GBM’s agential capacities remains a challenge, including, as our data demonstrate, at the individual level. Importantly, that pleasures and harms can exist concurrently does not mean that they always exist in equal measure. However, the dichotomous polarizing narratives that commonly shape thinking on substances make it difficult to attend to, as one participant articulated it, the ‘grey areas’ of AODC – a ‘grey area’ which is biopolitically shaped by normative cultural expectations for GBM to use substances to forge identity and have sex, and oppositional health discourses which position AODC as a significant threat to GBM’s mental health and wellbeing. Our attention to the ‘grey area’ is not an attempt to widen the pathologizing gaze on GBM’s drug-taking practices or an effort to illicit further self-pathologization. Rather, it is a call to move past restrictive binary frames in order to take seriously GBM’s vocalised concerns, desires, uncertainties, curiosities, expectations, pleasures and pains regarding the complex interactions between their wellbeing and their substance taking. Rather than *a priori* considering certain substance taking practices as clear and sole consequences of minority stress or trauma in need of some health intervention, or *a priori* determining certain substance taking practices as predominately indispensable to mental wellbeing, sex and GBM identity or community, centring the voices, needs and priorities of GBM when it comes to their *widely diverse* AODC practices, is imperative to creating effective community programming and support. The current hyper polarization of scientific and community discourse on GBM’s AODC does not appear to adequately capture the complexity of GBM’s lived experiences, and tends to either reinforce stigmatizing or pathologizing narratives about AODC, or, in a counter response to such stigma, minimizes some GBM’s stated concerns as being merely disciplinary responses to dominant heteropatriarchal biomedical discourse. However, to biopolitically frame AODC as *technologies of the self* is also to recognize that GBM are drawing from multiple expert and community discourses in ways that have, as was evident in the data, material effects on their health, wellbeing and social relationships. Understanding when and why our current community, scientific, policy and service discourses are succeeding or failing to succeed at actually centring everyday GBM’s needs is thus essential to the biopolitical goal of fostering the life this community.

This qualitative study is limited by having a modest sample size of English speaking GBM living within a major metropolitan centre. From the data, it is difficult to determine how experiences with crystal methamphetamine fundamentally differ from other drugs. Similarly, we cannot offer a comprehensive view of PnP’s effects on the mental health of GBM. Finally, it can be argued that the broader objectives of our study on mental health could have led participants to detail more negative versus positive accounts about AODC. Nonetheless, our sample did have a fairly significant range of mental health experiences and many participants spoke about the positive attributes of AODC.

## Conclusion

Despite cultural perceptions of substance use as prevalent and more accepted within GBM communities, some GBM can be highly conflicted about their AODC and ambivalent about its effects on their mental health and wellbeing. There is a need for harm reduction and AODC services capable of addressing this community’s concerns. Our participants’ accounts demonstrate that existing services and education are currently inadequate for meeting their needs, though some GBM struggling with their substance use actively seek such services. Rather than merely expanding upon existing service options, significant consultation with GBM who use substances as well as service providers working with this population are needed in order to innovatively improve upon the types of supports available in Ontario and ensure that available options do not continue to pathologize GBM. We also recommend community consultations focussing on how to effectively move past polarizing AODC discourse. Participants’ reported reluctance to participate in events held in person and the fact that many would not view their AODC as ‘problematic’, indicates a need for more online resources about AODC that can be accessed independently and designed to encourage reflexivity across the full spectrum of AODC experiences. Currently, very few online resources about AODC exist in Canada targeted to the GBM community, though a mental health resource our research team made (www.goodhead.ca) does begin some of this work, and the Gay Men’s Sexual Health Alliance (GMSH) in Ontario is currently developing a PnP resource and doing additional community-based research in this area. Perhaps what our data make most clear, however, is that whatever choices GBM are making in relation to their substance taking, they are exhibiting complex forms of agency. Centring such agency may be key to constructing community-level education and programming able to resonate with these men and empower them in their health decision-making.
